# Identification of Migratory Insects from their Physical Features using a Decision-Tree Support Vector Machine and its Application to Radar Entomology

**DOI:** 10.1038/s41598-018-23825-1

**Published:** 2018-04-03

**Authors:** Cheng Hu, Shaoyang Kong, Rui Wang, Teng Long, Xiaowei Fu

**Affiliations:** 1 Radar Research Lab, School of Information and Electronics, Beijing Institute of Technology, Beijing, 100081 China; 2 Key Laboratory of Electronic and Information Technology in Satellite Navigation (Beijing Institute of Technology), Ministry of Education, Beijing, 100081 China; 3 State Key Laboratory for Biology of Plant Diseases and Insect Pests, Institute of Plant Protection, Chinese Academe of Agricultural Sciences, Beijing, 100193 China; 4 Department of Electronic Engineering, Tsinghua University, Beijing, 100084 China

## Abstract

Migration is a key process in the population dynamics of numerous insect species, including many that are pests or vectors of disease. Identification of insect migrants is critically important to studies of insect migration. Radar is an effective means of monitoring nocturnal insect migrants. However, species identification of migrating insects is often unachievable with current radar technology. Special-purpose entomological radar can measure radar cross-sections (RCSs) from which the insect mass, wingbeat frequency and body length-to-width ratio (a measure of morphological form) can be estimated. These features may be valuable for species identification. This paper explores the identification of insect migrants based on the mass, wingbeat frequency and length-to-width ratio, and body length is also introduced to assess the benefit of adding another variable. A total of 23 species of migratory insects captured by a searchlight trap are used to develop a classification model based on decision-tree support vector machine method. The results reveal that the identification accuracy exceeds 80% for all species if the mass, wingbeat frequency and length-to-width ratio are utilized, and the addition of body length is shown to further increase accuracy. It is also shown that improving the precision of the measurements leads to increased identification accuracy.

## Introduction

Many organisms of numerous taxa migrate in the lower atmosphere. Among these aerial migrants, insects, which represent largest quantity of species and have abundant biodiversity, influence population dynamics, provide ecosystem services, spread plant and zoonotic diseases and cause sudden outbreaks of crop pests^1–3^. Long-distance migration is indispensable in the life-cycle of many insect species^4^. Effective monitoring of insect migration is extremely important to the study of ‘Migration Entomology’, because it reveals the behavioral adaptations that facilitate these movements and contributes to understanding how migration events change with climatic cycles^5,6^. However, most insects are too small for visual observation and may fly at night, at hundreds of meters above the ground, making it difficult to track them. Radar is the most effective tool for monitoring insect migration because it can directly detect migrating insects without perturbing them. Since the 1960s, radar has been applied to study the migration processes and phenomena of insects^7^. Several significant contributions have been made in applications of current entomological radar such as with respect to insect layering, navigation, wind-related orientation and collective orientation behaviors^8–11^.

Current zenith-pointing linear-polarized small-angle conical-scan (ZLC) entomological radars have the capabilities of mass measurement and wingbeat frequency retrieval^12,13^, which have the potential to facilitate identification of migratory insects^4^. The mass retrieval of migratory insects based on radar cross-sections (RCSs) has long been studied with the objective of achieving improved accuracies^14,15^. The backscattered signal amplitude modulation induced by wing beating has been used to extract the wingbeat frequency for many years^13,16^; however, for some targets, a frequency cannot be retrieved. Recent research has demonstrated that the mass estimated from RCSs and the wingbeat frequency estimated using the micro-Doppler effect have uncertainty levels of ~±40% and ~±1 Hz, respectively^14,17^. In addition, a linear relationship has been observed between the radar shape (see ref.^14^ for details) and the target shape parameter (e.g., the body length-to-width ratio)^14^, which can be expected to provide further information on the target’s identity. Body length is also widely used in insect size characterization. However, there is no indication that the body length can be measured with radar, although it is likely to be strongly correlated with mass. In summary, the mass, wingbeat frequency and length-to-width ratio are the radar-derived parameters available for making identifications of species undertaking migrations.

Previous studies of insect species migration and identification differentiated locusts from most other migrant species based on their characteristics (size, shape, and wing beating) and trajectory information (speed, direction, and orientation) obtained via ZLC radars^12^. Moreover, it was demonstrated that insect classification can be divided into broad taxon classes based on the mass and RCS shape parameters^18^. However, to date, little progress has been reported in the automatic multi-class identification of insect migrants based on entomological radar observations. The identification of different insect species is a typical multi-class classification task and can be solved by various machine learning algorithms. Previous research identified 35 species of moths based on support vector machine (SVM)^19^ and four other methods (Bayes, instance-based learning, decision trees and random forests)^20^, and showed that that the SVM algorithm produced the best identification results^21^.

In this paper, we adopted a new formulation of the SVM algorithm known as the decision-tree SVM (DTSVM) to classify different insect migrants based on their physical features. This method can objectively analyze the space between different classes using class separability^22^. Twenty-three species of migratory insects captured by a searchlight trap were included in the study. The parameters (mass, wingbeat frequency and length-to-width ratio) of these species were measured and used to construct the classification model, after which the body length was also introduced to provide an additional dimension for discriminating between different target species. Additionally, the identification accuracy was constrained based on the measurement precision of entomological radars in actual applications; therefore, the relationship between the identification accuracy and the measurement precision of these parameters was also analyzed. These findings provide new insights for future radar applications of the species identification of migratory insects.

## Results

### Materials

A ground-based vertical-pointing searchlight trap equipped with a 1,000 W metal-halide lamp, which was used for sampling high altitude migrating insects up to 500 m above ground level, was placed on top of a house that was 8 m above sea level on Beihuang Island (38°40′N, 120°93′E)^23^. Beihuang, an island located at the center of the Bohai Gulf in northern China, is located in a migration corridor between Shandong Peninsula and Liaoning Peninsula^24,25^, and 119 species of migratory insects have been documented in this place, such as *Loxostege sticticalis* (Lepidoptera: Pyralidae), *Helicoverpa armigera* (Lepidoptera: Noctuidae) and *Spodoptera exigua* (Lepidoptera: Noctuidae), which are serious crop pests in northern China and have been studied in detail for many years^26–28^. The trapping process was conducted on all fair nights from August to October 2015, except during periods of rain or power outage. The attracted insects were captured with a sweep net and subsequently identified by species. The trapped individual insects that were not in sufficiently good condition to fly were discarded, and the species with insufficient specimen numbers were excluded from the analysis. Ultimately, 5532 individual migratory insects belonging to 23 species remained.

**Figure 1:**
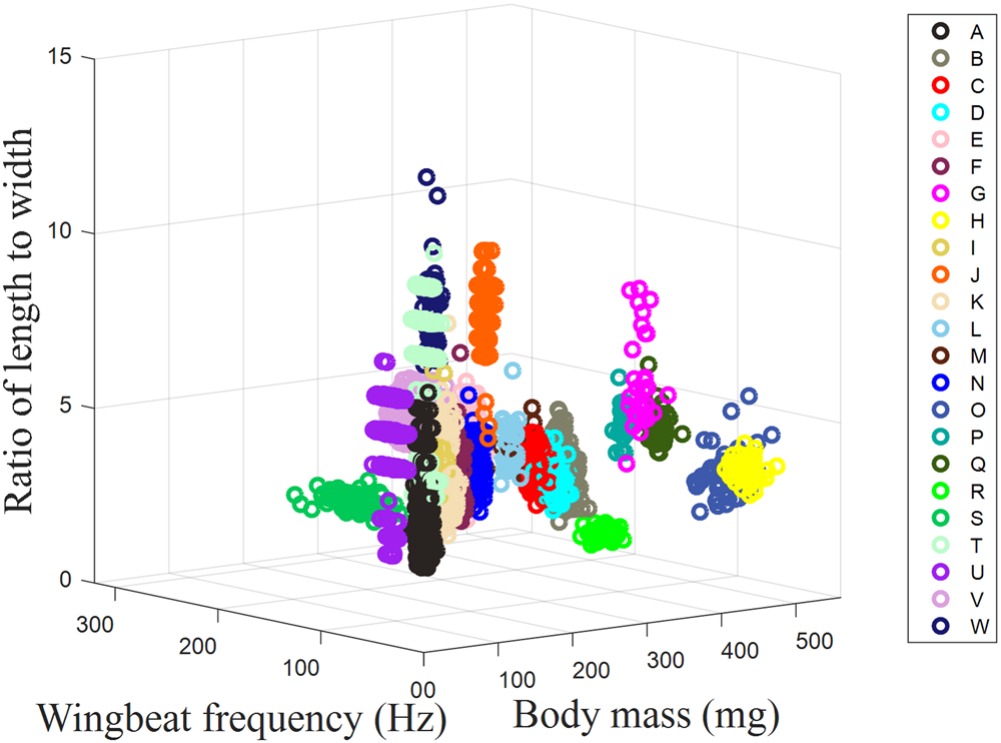
Distributions of the mass, wingbeat frequency and length-to-width ratio of all trapped insect samples. Note: each circle corresponds to an insect sample, with the different colors representing different insect species.

The primary information on the 23 species is shown in Table 1. For later convenience, each species is identified using a distinguishing class label (an uppercase letter) and color (see Fig. 1), and hereinafter, the specific label and color are adopted to represent each species. The insect mass was measured using an electronic balance with measurement accuracy of 0.1 mg. The insect body length and width were measured using a steel rule with a minimum scale of 1 mm, and the length-to-width ratio was then also calculated. To measure the wingbeat frequency, individual insects were collected and were subsequently anaesthetized with ether. After that, a steel wire of about 10 cm length was glued to the back of each specimen which was then hoisted in an incubator^29^. In our experiments, the wingbeat frequency was measured by a stroboscope, and the steady flight was needed. As we know, when the insect’s feet are detached from a surface, it is supposed to start wing-beating spontaneously. However, insects sometimes keep wing-beating only for a very short time, so the wingbeat frequency can not be measured successfully by only one time. In our experiment, many times of measurements were carried out until the steady flight happened, and then the wingbeat frequency was measured. In addition, some insects always failed to commence wing-beating or to keep steady flight, so these insects were discarded ultimately. For the steady flight of insects, the wingbeat frequency was measured using a stroboscope with a measurement accuracy of 0.01%, and in this case, the minimum precision was 1 Hz. It is important to note that the wingbeat frequency can be affected by the flying status and the ambient environment; therefore, some uncertainty exists in the measurement of the wingbeat frequency. Previous research suggested that a correction of 15% should be applied to the frequencies of grasshoppers obtained by the ‘balloon-release’ method because laboratory experiments have shown that a decrease in frequency of 10–20% occurs during the first few minutes of flight^30,31^. However, the unmodified measurements have been used in this study.

**Table 1:** Primary information for the 23 insect species. Note: all captured insects were identified by Xiaowei Fu.

Label	Species	Family	Order	Quantity	Mass (mg)			Wingbeat frequency (Hz)			Length to width ratio			Body length (mm)		
					Range	Mean	Std. Dev.	Range	Mean	Std. Dev.	Range	Mean	Std. Dev.	Range	Mean	Std. Dev.
A	*Eriopyga grandis*	Noctuidae	Lepidoptera	473	55.7–69.3	62.98	2.41	36–57	45.15	3.57	2–7	3.52	0.91	8–14	11.22	1.12
B	*Agrotis tokionis*	Noctuidae	Lepidoptera	382	252.2–280.9	266.45	4.97	38–75	58.30	5.85	2.7–5.8	3.88	0.51	24–31	27.18	1.35
C	*Agrotis c-nigrum*	Noctuidae	Lepidoptera	43	203.2–224.7	215.01	5.38	36–57	46.44	5.41	3.3–5.4	4.53	0.57	21–27	24.47	1.47
D	*Agrotis praecox*	Noctuidae	Lepidoptera	78	209.9–253.3	233.83	7.86	31–58	42.47	5.38	3.2–5.2	4.00	0.46	18–27	22.70	1.83
E	*Spodoptera litura*	Noctuidae	Lepidoptera	129	37.1–152.0	143.97	2.59	45–66	56.88	3.98	3–7	4.93	0.80	15–21	18.64	1.35
F	*Heliothis dipsacea*	Noctuidae	Lepidoptera	84	100.6–114.4	107.37	2.72	37–56	44.31	3.48	3.2–8.0	4.44	0.84	13–19	15.51	1.26
G	*Speiredonia retorta*	Noctuidae	Lepidoptera	32	306.1–355.6	326.96	10.63	18–30	24.75	3.02	4.4–9.3	6.72	1.32	22–29	25.47	1.88
H	*Dermaleipa juno*	Noctuidae	Lepidoptera	61	444.5–511.8	472.79	13.09	22–34	28.34	2.48	3.0–4.5	3.71	0.30	39–45	42.56	1.47
I	*Acronicta rumicis*	Noctuidae	Lepidoptera	58	76.7–91.8	85.35	4.18	41–57	48.76	3.92	4.0–7.5	4.99	0.56	12–16	14.72	0.87
J	*Calospilos suspecta*	Geometridae	Lepidoptera	147	110.9–127.8	117.43	3.04	19–33	25.97	2.84	5.6–11	9.31	0.87	16–22	18.78	1.39
K	*Spilarctia subcarnea*	Arctiidae	Lepidoptera	296	85.6–104.7	94.60	3.54	33–64	47.78	5.10	2.8–8.9	4.30	0.82	15–21	18.10	1.11
L	*Spilosoma niveus*	Arctiidae	Lepidoptera	48	178.3–198.3	188.20	4.35	42–66	52.94	4.19	4.0–7.2	5.07	0.61	24–29	27.38	1.20
M	*Amsacta lactinea*	Arctiidae	Lepidoptera	27	169.0–242.7	218.71	17.49	50–63	57.00	2.95	4–6	4.76	0.51	22–28	24.81	1.18
N	*Rhyparioides amurensis*	Arctiidae	Lepidoptera	144	125.0–145.2	134.30	3.68	39–53	46.27	2.95	3.4–6.7	4.49	0.63	14–20	16.77	1.20
O	*Clanis bilineata*	Sphingidae	Lepidoptera	53	416.0–519.0	459.28	23.32	31–40	36.51	1.76	2.6–5.8	3.56	0.62	38–47	42.98	2.18
P	*Psilogramma menephron*	Sphingidae	Lepidoptera	29	311.4–337.5	326.18	5.82	38–45	41.48	1.62	4.5–6.7	5.43	0.49	41–48	46.00	1.87
Q	*Ampelophaga rubiginosa*	Sphingidae	Lepidoptera	41	365.2–412.6	381.99	8.60	44–54	48.46	1.95	4.4–6.9	5.10	0.46	43–49	46.85	1.37
R	*Callambulyx tartarunovii*	Sphingidae	Lepidoptera	35	277.0–332.7	304.78	13.68	42–50	45.60	1.93	2.0–2.6	2.26	0.17	28–34	31.29	1.34
S	*Macroglossum stellatarum*	Sphingidae	Lepidoptera	84	211.7–244.1	227.29	7.00	182–289	233.40	19.60	1.8–2.9	2.25	0.19	24–31	27.77	1.47
T	*Loxostege sticticalis*	Pyralididae	Lepidoptera	892	59.9–75.3	67.37	2.39	33–60	47.21	4.35	3.0–11	7.11	2.19	6–11	8.71	0.66
U	*Spoladea recurvalis*	Pyralididae	Lepidoptera	1574	5.2–23.8	14.52	2.79	28–59	42.28	4.88	2.5–8.0	6.14	0.71	4–8	6.19	0.58
V	*Pantala flavescens*	Libellulidae	Odonata	768	138.8–223.0	182.77	13.07	120–155	137.48	5.70	4.4–6.5	5.40	0.37	60–74	66.64	2.26
W	*Enallagma cyathigerum*	Coenagriidae	Odonata	54	121.1–130.1	125.15	2.05	74–92	82.76	3.87	7.3–13	9.04	1.07	42–53	47.19	1.91

**Figure 2:**
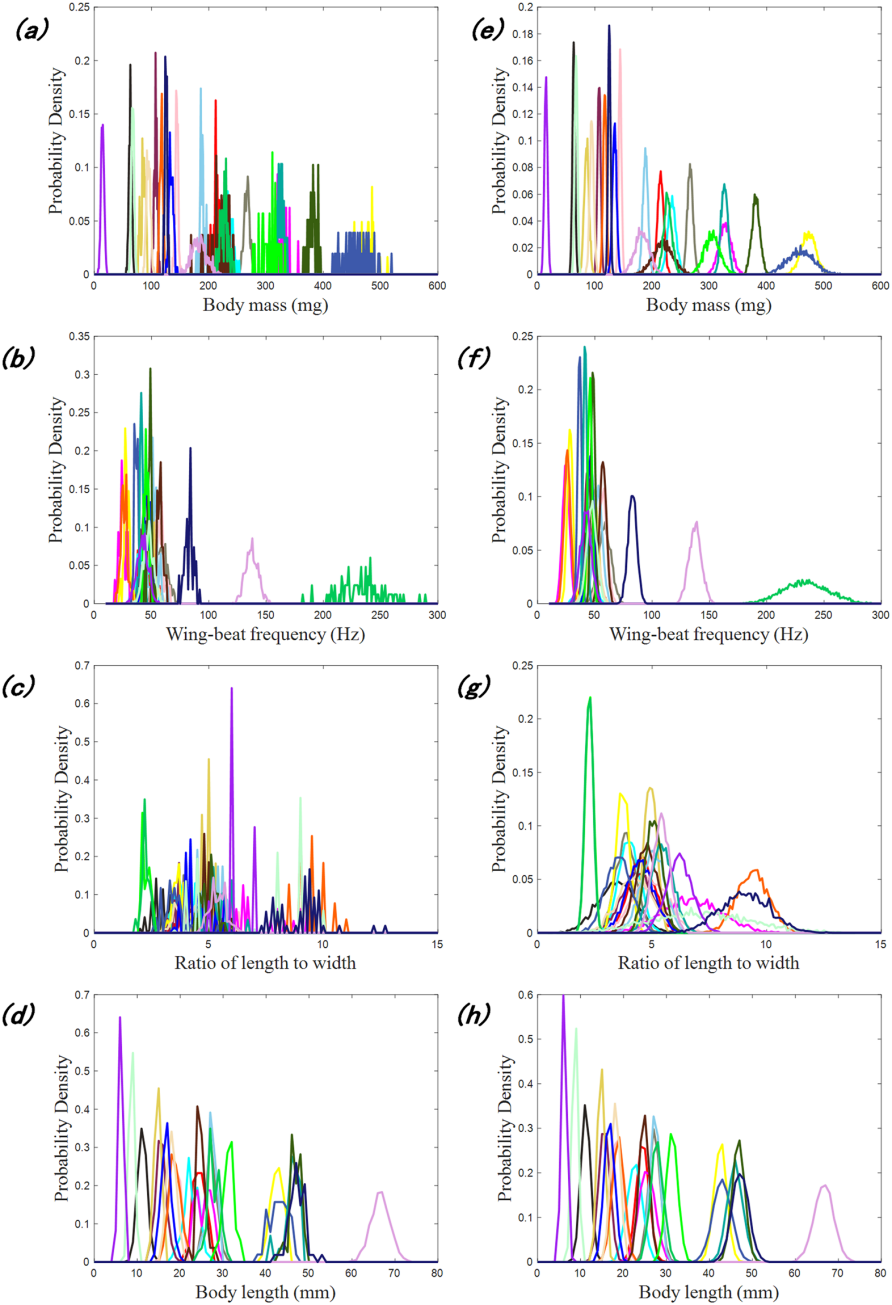
Probability distribution curves of the mass, wingbeat frequency, length-to-width ratio and body length of all trapped species: (**a**–**d**) original samples and (**e**–**h**) constructed samples.

The mass, wingbeat frequency, length-to-width ratio and body length ranged from 5.2 mg to 519 mg, 18 Hz to 289 Hz, 1.8 to 13 and 4 mm to 74 mm, respectively. The distributions of the mass, wingbeat frequency, and length-to-width ratio for all insect samples are shown in Fig. 1. A large set of sample data is necessary to construct a classification model with high accuracy and robustness. The sample quantities of these species range from 29 to 1547, and certain species (C, G, L, M, P, Q and R) include less than 50 specimens. Thus, an extension of the original sample data for these species was conducted in our research. In the extension, the mean, standard deviation, and covariance of each species were calculated, and a new dataset was generated using these statistical parameters based on the normal distribution. Finally, each species was extended to 5,000 samples. For comparison, the original samples and the constructed samples are shown in Fig. 2, where Fig. 2(a–d) correspond to the original samples, and Fig. 2(e–h) correspond to the constructed samples. After the extension, the probability distribution curves appear to satisfy the normal distribution, especially (as is to be expected) for species with a small original sample set. Next, the constructed dataset was utilized to build the classification model of the 23 insect species. The extension samples of each species were equally divided into two datasets for training and testing.

### Separability analysis of different features

**Figure 3:**
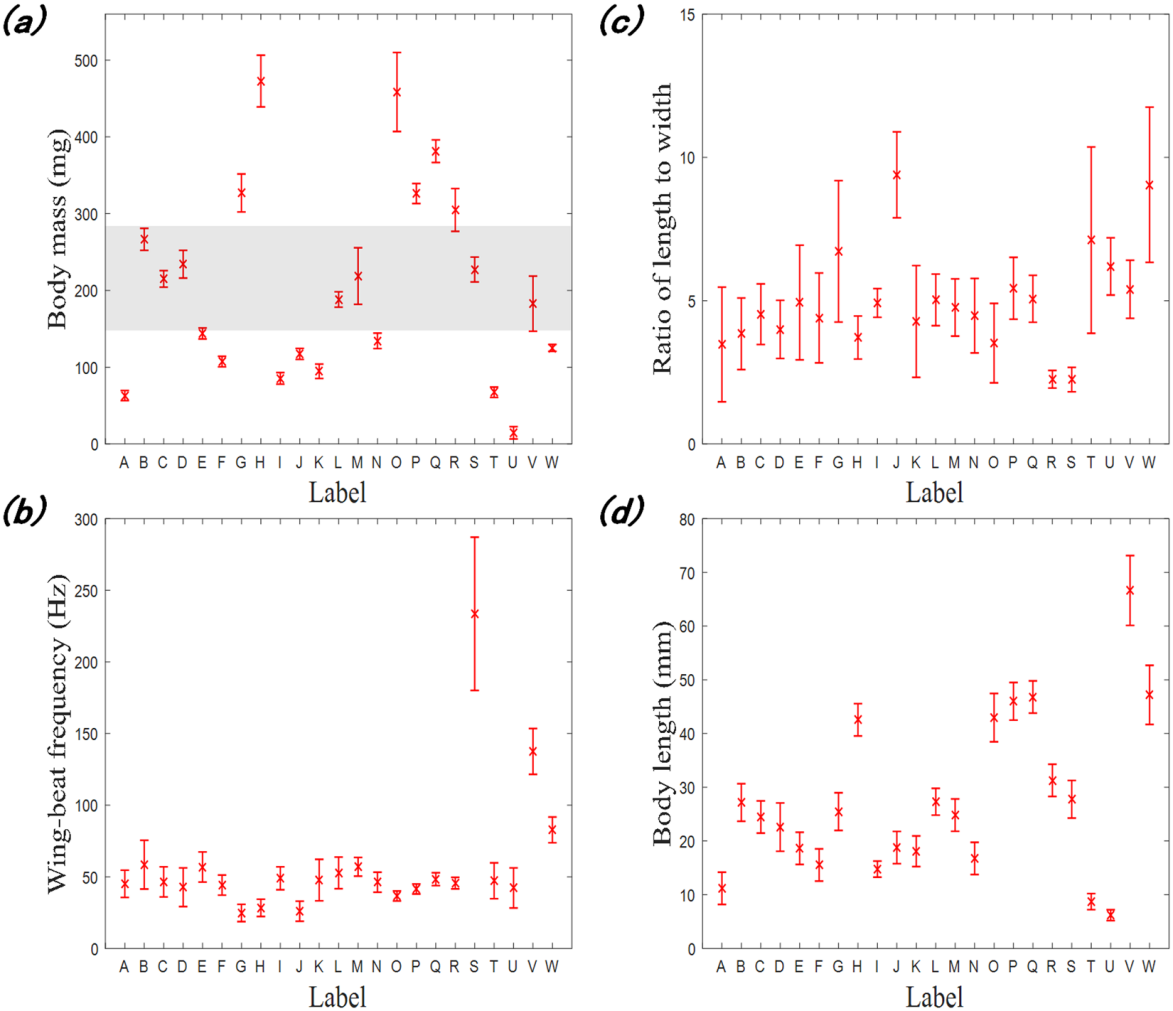
Distributions of the physical features of all trapped species.

**Figure 4:**
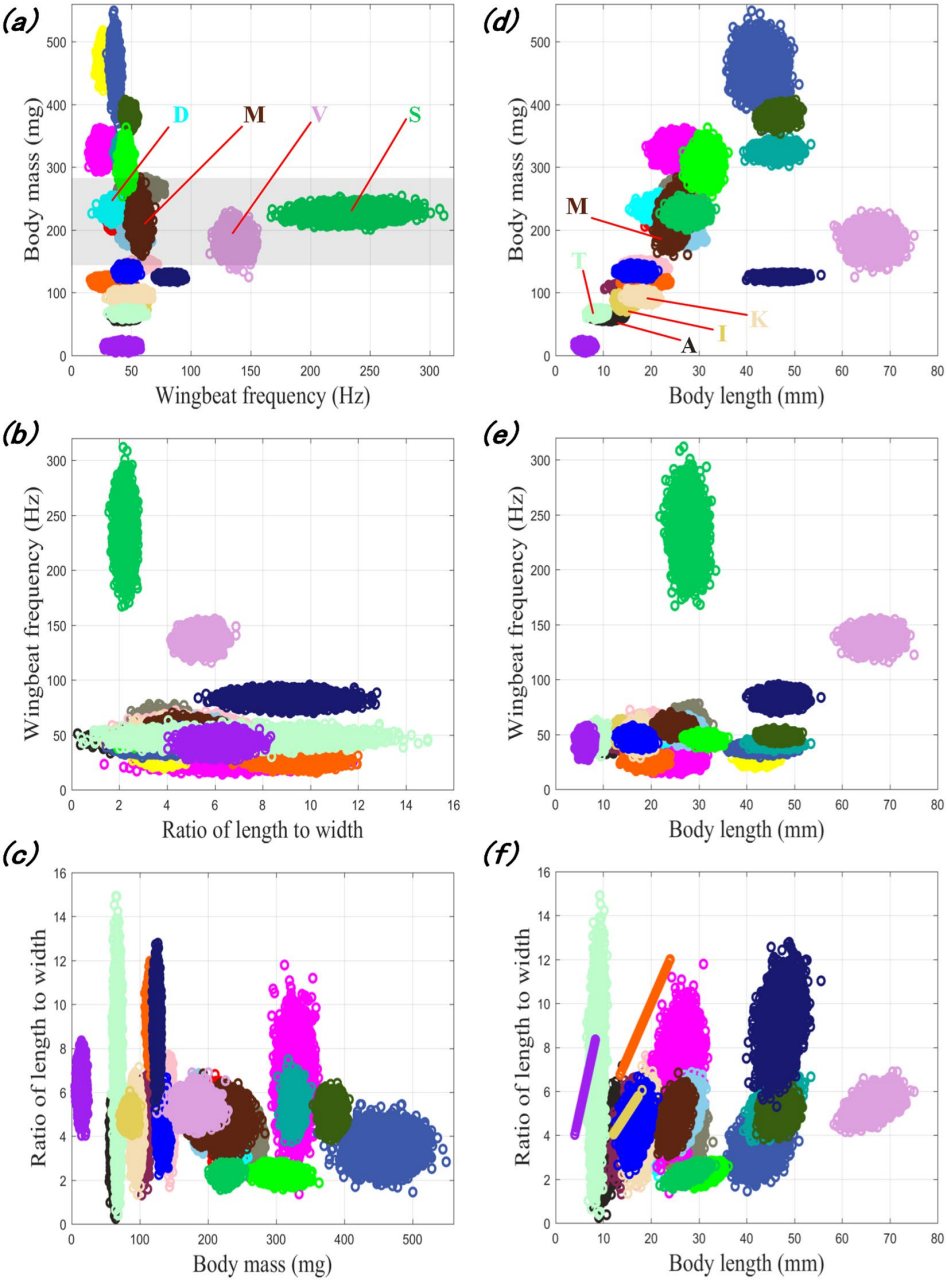
Pairwise correlations of the physical features of all trapped species. Note: The linear distributions in (**f**) arise because all specimens of these species had identical width measurements.

For intuitive analysis, the distributions of the mass, wingbeat frequency, length-to-width ratio and body length of all trapped insects are given in Fig. 3, where the X-axis represents different insect species, and the Y-axis represents the physical features. Each line segment in Fig. 3 indicates the median and min-max range of the feature parameter. The wingbeat frequency of most species ranges from 20 Hz to 80 Hz and it is evident that the overlap is severe, whereas the ranges of the mass and body length are narrow, which could improve separability. A better discrimination of different species can be observed from the distributions of the two-dimensional features in Fig. 4. For instance, as shown in Fig. 4(a), the mass of certain species overlaps in the range of 150 mg to 350 mg, but a subset of these species (e.g., M, V, and S) can be identified by including the wingbeat frequency in the identification process (Fig. 4(a)). To identify as many insect species as possible with high recognition rates, it is better to combine additional features. The mass, wingbeat frequency and length-to-width ratio can be retrieved by existing entomological radar; therefore, we constructed a classification model based on these three features. Although the mass and body length appear to be strongly correlated, as shown in Fig. 4(d), the body length was included to assess its contribution to the identification as an extra variable. A detailed analysis is given in the following sections.

### Species identification of migratory insects

**Figure 5:**
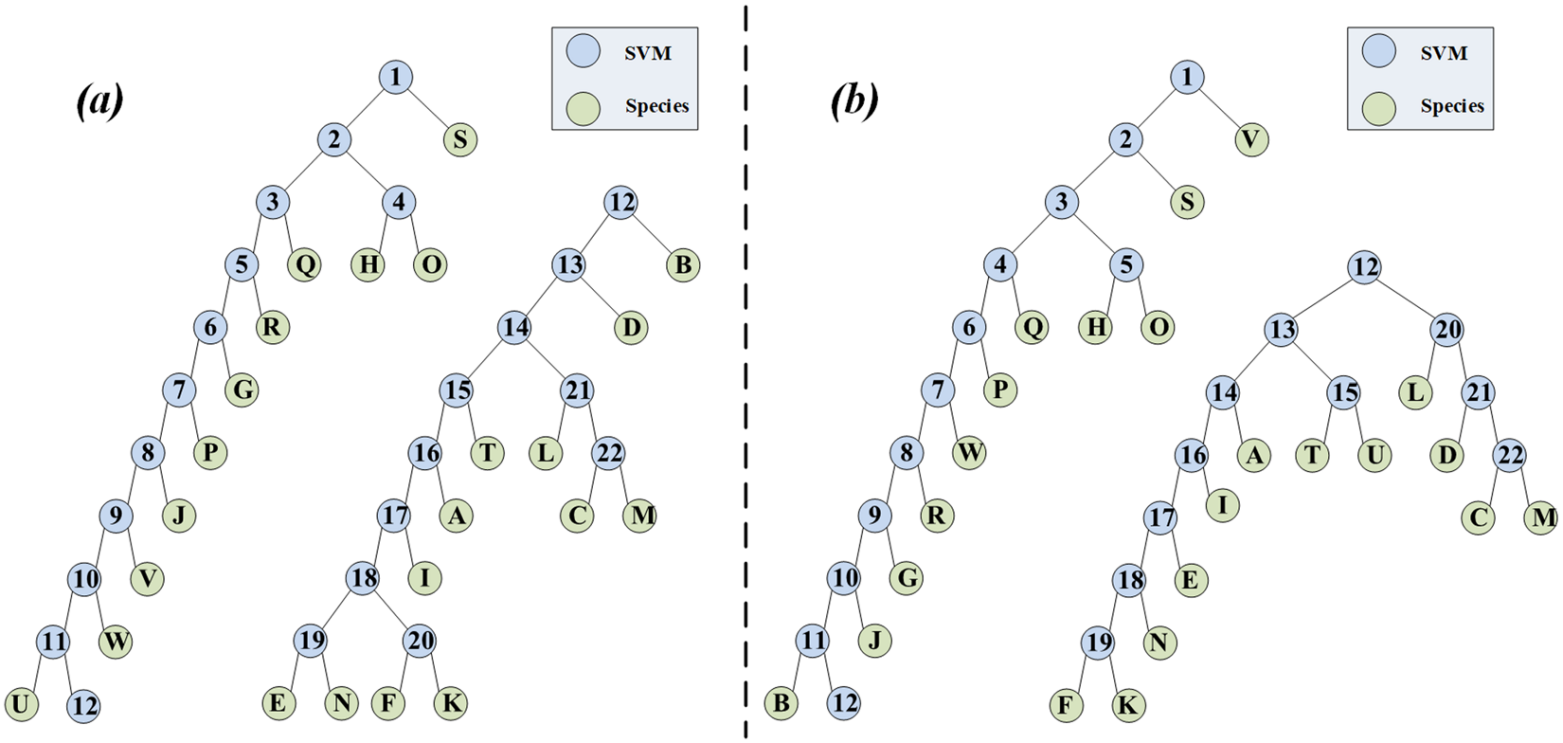
Classification schemes: (**a**) based on the mass, wingbeat frequency and length-to-width ratio; (**b**) based on the mass, wingbeat frequency, length-to-width ratio and body length. Note: The blue circles with numbers represent SVM classifiers, and the green circles with uppercase letters represent insect species. For a better figure arrangement, the subsequent classifiers after SVM 12 are placed on the right in (**a**), and a similar arrangement is used in (**b**).

First, the mass, wingbeat frequency and length-to-width ratio were used to identify the 23 trapped species. The classification scheme is shown in Fig. 5(a), where the multi-class classification problem is converted into several binary classification problems and SVM is used to develop a classifier for each binary classification problem. The cascade sequences of these binary classifiers are automatically built based on the separability factor (refer to Equation (1) in the Method section). Each SVM classifier is trained and determined based on the training samples. Figure 5(b) gives the classification scheme based on the mass, wingbeat frequency, length-to-width ratio and body length. The binary trees become noticeably different when the body length is introduced. See the Methods section for the details of the scheme construction.

After all classifiers are trained with training samples, the testing samples are used to assess the classification performance, and the results are given in Table 2. For the classification scheme based on the mass, wingbeat frequency and length-to-width ratio, the identification accuracy ranges from 84% to 100% with a mean value of 97%. The high identification accuracies indicate that the proposed classification scheme based on the mass, wingbeat frequency and length-to-width ratio is useful and robust in the identification of the 23 trapped species. For the classification scheme based on the mass, wingbeat frequency, length-to-width ratio and body length, the identification accuracies of all species are improved, with species A, I, K, M and T particularly benefitting from inclusion of the additional parameter; specifically, the mean identification accuracy increases to 98%. Therefore, the body length can also be considered as a feature for species identification.

**Table 2:** Identification accuracy of all trapped species.

Label	Identification accuracy	
	Classification scheme based on the mass, wingbeat frequency and length-to-width ratio	Classification scheme based on the mass, wingbeat frequency, length-to-width ratio and body length
A	0.96	0.99
B	0.99	0.99
C	0.88	0.89
D	0.92	0.93
E	0.98	0.99
F	0.98	1.00
G	1.00	1.00
H	0.98	0.98
I	0.92	1.00
J	1.00	1.00
K	0.92	0.99
L	0.96	0.98
M	0.85	0.88
N	0.99	0.99
O	0.98	0.99
P	1.00	1.00
Q	1.00	1.00
R	1.00	1.00
S	1.00	1.00
T	0.92	0.99
U	1.00	1.00
V	1.00	1.00
W	1.00	1.00
**Mean value**	0.97	0.98

### Measurement precision requirements

The measurement precision of the mass, wingbeat frequency, length-to-width ratio and body length is high for the trapped insects, because these measurements were made indoors with high-precision instruments. Unfortunately, both the precision of radar measurements of the insect RCSs and the retrieval accuracy of the physical features based on laboratory-measured RCSs are not yet of this sufficient. Therefore, the identification performance was evaluated under different measurement precisions to offer an indicator for the entomological radar when it is applied in species identification of migratory insects.

Different measurement errors were introduced in the training and testing samples of the 23 insect species. Four different errors were considered, as listed in Table 3, where the standard deviations of the wingbeat frequency measurement errors are all 1 Hz. In Case I, the mass, wingbeat frequency and length-to-width ratio are used in the classification. The root mean square percent error (RMSPE) of the mass is set to 40%, which is consistent with the current retrieval accuracies under laboratory conditions, and that of the length-to-width ratio is also set to 40%. Case II and Case III demonstrate the identification accuracy improvement if the RMSPE values of both the mass and length-to-width ratio decrease from 40% to 20% and subsequently to 10%. In Case IV, the body length is introduced as an extra variable to improve the identification performance, assuming that the body length can be retrieved with an RMSPE of 10%.

**Table 3:** Four cases of measurement errors. Note: the RMSPE statistic is generally used to assess the deviations between the observed values and true values^36^.

Error label	Standard deviation	Root mean square percent error		
	Wingbeat frequency (Hz)	Mass	Length-to-width ratio	Body length
Case I	1	40%	40%	—
Case II	1	20%	20%	—
Case III	1	10%	10%	—
Case IV	1	10%	10%	10%

The testing samples with different measurement errors were processed using the classification scheme that was trained with the corresponding training samples, and the classification results are listed in Table 4. For Case I, the mean identification accuracy is 56%, which implies that the current entomological radar has low identification performance. However, when the RMSPEs of the mass and length-to-width ratio decrease from 40% to 20% and subsequently to 10%, the mean identification accuracy increases to 71% and subsequently to 84%. Additionally, the introduction of the body length with an RMSPE of 10% further improves the identification performance, with a mean identification accuracy of 88%. Therefore, improving the mass and length-to-width ratio measurements could significantly contribute to the identification performance, and the introduction of an extra variable could improve it further.

**Table 4:** Identification accuracy of all trapped species based on different measurement errors.

Label	Identification accuracy			
	Classification scheme based on the mass, wingbeat frequency and length-to-width ratio			Classification scheme based on the mass, wingbeat frequency, length-to-width ratio and body length
	Case I	Case II	Case III	Case IV
A	0.43	0.79	0.88	0.95
B	0.51	0.68	0.84	0.86
C	0.17	0.27	0.56	0.60
D	0.20	0.53	0.65	0.70
E	0.45	0.60	0.79	0.92
F	0.53	0.53	0.72	0.75
G	0.65	0.89	0.98	1.00
H	0.83	0.91	0.96	0.96
I	0.35	0.54	0.66	0.78
J	0.95	0.99	1.00	1.00
K	0.23	0.28	0.52	0.75
L	0.07	0.27	0.60	0.70
M	0.46	0.49	0.67	0.69
N	0.22	0.44	0.77	0.80
O	0.77	0.90	0.96	0.97
P	0.53	0.76	0.91	0.97
Q	0.65	0.87	0.97	0.97
R	0.55	0.91	0.98	0.99
S	1.00	1.00	1.00	1.00
T	0.42	0.67	0.82	0.94
U	0.86	1.00	1.00	1.00
V	1.00	1.00	1.00	1.00
W	1.00	1.00	1.00	1.00
**Mean value**	0.56	0.71	0.84	0.88

## Discussion

This study demonstrates that the mass, wingbeat frequency and length-to-width ratio, as well as the body length, can be utilized to identify insects with high accuracy using the DTSVM method. By analyzing the identification accuracy for different measurement errors, we found that improving the measurement precisions of the mass and length-to-width ratio can greatly facilitate the identification of migratory insects; therefore, the radar measurement precision of insect RCSs and the precision of feature retrieval based on laboratory-measured RCSs must be further improved to achieve better identification. In addition, current entomological radars are unable to measure the body length, which restricts further improvement in the identification performance. Therefore, development of new radar techniques that can measure the body length, or some other additional characteristics of the target, would be beneficial.

Furthermore, these four features might be unstable and vary with the ambient environment (e.g., air temperature affecting wing-beat frequency) and biological factors (e.g., age and the conditions in which the insects developed). These issues complicate insect identification and requires further investigation^12^. One reason for why the ranges of the mass and body length are narrow might be that the insects were trapped in the same place from a single migration corridor: it is highly likely that the samples consist of individuals undertaking migration, and thus exhibit a uniform physiological condition. For application at other sites, similar analyses of samples from those sites might be required.

The classification schemes proposed here have been developed for the 23 migratory species captured in the Bohai Gulf and are successful for these species. However, when more diverse fauna are considered, the method may exhibit higher rates of incorrect identification. Realistically, for a certain site during a particular season, the number of major migratory insect species is usually small, and other migratory pests may be present only in small numbers, so that there is no need to consider them. Thus, there is no need to consider these species. If an additional insect species becomes common at the observation site, it can be incorrectly identified. Thus, a category that does not fit any of the training classes is needed, a topic that should be fully investigated in the future.

## Methods

### Classification method

SVM was originally designed as a binary classifier, and it can be applied to multi-class classification problems when combined with a decision tree. DTSVM can avoid error accumulation in the training and modeling processes^32^. The process of constructing an identification model can be divided into the following two steps. The first step is a modeling step, in which a binary tree model is constructed by converting the multi-class classification problem into several binary classification problems. The second step is a training step in which SVM is used to train each binary classifier and obtain a predictive model that links the parameters to the labels.

### Construction of a binary tree model

To construct a classification model, we must convert an N-class classification problem into a sequence of N-1 two-class classification problems. The classification model is a binary tree with N-1 nodes and n leaf nodes. Each node represents a binary SVM classifier, and each leaf node represents one class (Fig. 5). The hierarchical structure of the model depends on the separability of two subclasses. The classification performance of models with different hierarchical structures greatly differs, and therefore, a reasonable hierarchical structure is critically important. To establish an effective structure, a reasonable inter-class separability measure must be defined for the two classes.

Consider a dataset $\{({{\boldsymbol{x}}}_{1},{y}_{1}),\ldots ,({{\boldsymbol{x}}}_{l},{y}_{l})\}$, ${{\boldsymbol{x}}}_{{\boldsymbol{i}}}\in {R}^{n},i=1,\ldots \,l$, and ${y}_{i}$ is the associated class label. To evaluate the separability of different species, the sample center ${{\bf{m}}}_{i}$ of each class is calculated via the K-means method^33^. Thus, the inter-class separability factor (ICSF) of class $i$ and class $j$ can be defined as follows^34^:

1 $$d{m}_{ij}=\frac{d({{\bf{m}}}_{i},{{\bf{m}}}_{j})}{({\sigma }_{i}+{\sigma }_{j})},i,j=1,\,\ldots \,l$$

where $d({{\bf{m}}}_{i},{{\bf{m}}}_{j})$ represents the Euclidean distance between the sample centers of class $i$ and class $j$ and ${{\sigma }_{i}}^{2}$ represents the sample variance of class $i$.

The misidentification rate of two subclasses decreases as $d{m}_{ij}$ increases, and the subclasses with the largest ICSF are the most separable. To avoid error propagation, the node with the lowest misidentification rate should be placed at the top of the binary tree. The process of constructing a binary tree is described as follows.

Step 1: Using the training dataset, compute the ICSF of each pair of known subclasses according to Eq. (1). Find the smallest value of the ICSF, and merge the two corresponding subclasses into one. Note that the two subclasses with the smallest ICSFs are the most inseparable.

Step 2: Repeat step 1 with the new and smaller set of classes. Continue, constructing a binary tree, until all classes have been combined.

Step 3: Reverse the tree such that subclasses that are most separable are placed at the top of the tree. After this step, the multi-class classification model is thus built.

### Support vector machine

SVM is a machine-learning technique that has been successfully applied in several domains including insect identification. The library of Support vector machines (LIBSVM^35^), one of the most widely used pieces of SVM software packages, is used to solve each two-class classification problem.

### Data availability

The datasets analyzed in this study are available from the corresponding author upon reasonable request.
